# The effectiveness of recruitment strategies on general practitioner’s survey response rates – a systematic review

**DOI:** 10.1186/1471-2288-14-76

**Published:** 2014-06-06

**Authors:** Sabrina Winona Pit, Tham Vo, Sagun Pyakurel

**Affiliations:** 1 University Centre for Rural Health, University of Sydney, Uralba Street, Lismore NSW 2480, Australia

## Abstract

**Background:**

Low survey response rates in general practice are common and lead to loss of power, selection bias, unexpected budgetary constraints and time delays in research projects.

**Methods:**

Objective: To assess the effectiveness of recruitment strategies aimed at increasing survey response rates among GPs.

Design: Systematic review.

Search methods: MEDLINE (OVIDSP, 1948-2012), EMBASE (OVIDSP, 1980-2012), Evidence Based Medicine Reviews (OVIDSP, 2012) and references of included papers were searched. Major search terms included GPs, recruitment strategies, response rates, and randomised controlled trials (RCT).

Selection criteria: Cluster RCTs, RCTs and factorial trial designs that evaluate recruitment strategies aimed at increasing GP survey response rates.

Data collection and analysis: Abstracts identified by the search strategy were reviewed and relevant articles were retrieved. Each full-text publication was examined to determine whether it met the predetermined inclusion criteria. Data extraction and study quality was assessed by using predetermined checklists.

**Results:**

Monetary and nonmonetary incentives were more effective than no incentive with monetary incentives having a slightly bigger effect than nonmonetary incentives. Large incentives were more effective than small incentives, as were upfront monetary incentives compared to promised monetary incentives. Postal surveys were more effective than telephone or email surveys. One study demonstrated that sequentially mixed mode (online survey followed by a paper survey with a reminder) was more effective than an online survey or the combination of an online and paper survey sent similtaneously in the first mail out. Pre-contact with a phonecall from a peer, personalised packages, sending mail on Friday, and using registered mail also increased response rates in single studies. Pre-contact by letter or postcard almost reached statistical signficance.

**Conclusions:**

GP survey response rates may improve by using the following strategies: monetary and nonmonetary incentives, larger incentives, upfront monetary incentives, postal surveys, pre-contact with a phonecall from a peer, personalised packages, sending mail on Friday, and using registered mail. Mail pre-contact may also improve response rates and have low costs. Improved reporting and further trials, including sequential mixed mode trials and social media, are required to determine the effectiveness of recruitment strategies on GPs' response rates to surveys.

## Background

GPs play an important role in the design and implementation of health services and health policy to improve the health outcomes of their patients [1]. Understanding and measuring GPs’ knowledge, attitudes, behaviours, practices and their views on solutions to health care issues are paramount to improve the quality of health care. Surveys are a useful tool to measure a wide variety of issues that are relevant to general practice. It has been argued that GPs are often time poor, are difficult to recruit for research studies [1,2] and often have low survey response rates [2] whereas the latter has been contended by others [3]. There has been a steady increase in research studies examining methods to increase response rates amongst doctors [3]. Despite the greater evidence base for improving response rates, a recent review of the primary care literature between 2000 and 2009 showed that GP response rates to postal surveys over the past decade are relatively unchanged [3]. The authors found that the average response rate was 61% (95% confidence interval (95% CI): 59% to 63%) amongst 371 GP surveys. Reasons for not participating in research are many, including concerns about disrupting routine practice [1,4], time [1,2,4], relevance of the study topic [1,2], confidentiality [1,2] and receiving many surveys a week. The general practice workforce is also increasingly working part-time [5]. Low response rates can lead to methodological biases, such as non-response bias, time delays in research projects, budgetary problems [6], underpowered studies and raise an ethical dilemma in that people have been subjected to research that can not show its effect [7].

Several systematic reviews have examined strategies amongst the general population to increase response rates to postal, electronic and telephone surveys [8,9]. Whilst general population reviews [7,8,10] are useful, it is likely that some strategies are more effective in increasing GP survey response rates than others. For example, GPs are probably less sensitive to monetary incentives than the general population given their higher income status. Many of the strategies applied to the general population have also been developed and tested to improve GP survey response rates such as monetary incentives [11], questionnaire length [12] and pre-contact [13]. Two systematic reviews [2,14] have examined how best to increase response rates amongst physicians. The first systematic review included studies conducted until 1999 and found that pre-notification, personalised mailouts and nonmonetary incentives were not associated with improved physician response to surveys [14]. On the contrary, monetary incentives, stamps and shorter surveys led to increased response rates. The second systematic review included studies until 2006 and broadly explored two intervention categories: incentives and design-based approaches. The authors found that even small financial incentives improved physician response rates but that token nonmonetary incentives were less effective. Postal and telephone surveys were more effective than fax and online surveys, as were mixed mode surveys, first class stamps, shorter surveys, personalised letters, and studies endorsed by reputable professional organisations [2]. The aim of this study was to assess the effectiveness of recruitment strategies aimed at increasing response rates of GPs to complete surveys.

## Methods

### Criteria for considering studies for this review

#### Types of studies

Cluster randomized controlled trials, randomized controlled trials and factorial trial designs that aimed to improve survey response rates amongst GPs.

#### Population

The primary population included GPs or family physicians.

#### Types of methods

Any intervention that compares different recruitment methods of GPs to complete surveys. Interventions aimed directly at patients were excluded. Studies that evaluated retention strategies were excluded. Interventions that recruited GPs for clinical trials were also excluded.

#### Types of outcome measures

The primary outcome measure was the proportion of eligible GPs who responded to surveys.

### Search methods

MEDLINE ( OVIDSP- 1948 to December 2012), EMBASE (OVIDSP- 1980 to December 2012), Evidence Based Medicine (EBM) Reviews (OvidSP - December 2012) and references of included papers, related literature and systematic reviews were searched. Major search terms included GPs, recruitment strategies, response rates and randomised controlled trials (RCT). The search strategy was built on previous searches [1,8,15,16]. The search strategy for Medline can be found in Additional file 1. Search results were merged into reference management software and duplicate records were removed.

### Data extraction

Abstracts identified by the search strategy were reviewed and relevant articles were retrieved. Titles and abstracts were independently checked by SP. A random number generator was used to select about 10% of the initial MEDLINE electronic citations. SP and SWP independently assessed 106 citations. Agreement of 99% was achieved and a Prevalence and Bias Adjusted Kappa Kappa of 0.98 (95% CI 0.79 to 1.17) [17]. Full-text papers were screened to determine eligibility by both SP and SWP. Disagreements were resolved by discussion. SP and TV extracted data. Ambiguities were resolved by discussion.

### Assessment of risk of bias in included studies

Two authors (SP and TV) independently assessed risk of bias using standard Cochrane criteria, including allocation concealment, random sequence generation, blinding of participants and personnel, complete outcome data and absence of reporting bias. Ambiguities were resolved by discussion. Intention-To-Treat analysis includes everyone who has been randomised to a treatment group, irrespective of participant’s non-compliance (eg not taking part in the treatment), protocol deviation or withdrawal from the study. Treated analysis is considered as high risk of complete outcome data bias, therefore the results of the assessment shows this element as a separate item for additional clarity. We considered absences of other bias in the form of whether differences in characteristics (eg age, gender) between study groups were reported.

### Data analysis

For each trial we calculated results in terms of odd ratios and their 95% CIs. Studies that were similar in terms of interventions were pooled by using Review Manager 5.2 software [18]. Heterogeneity was assessed with the I^2^ test and Q statistics. An I^2^ value greater than 50% and P < 0.1 was indicative of heterogeneity [19]. Random effects models were mainly used because of significant heterogeneity in some analyses.

## Results

**Figure 1:**
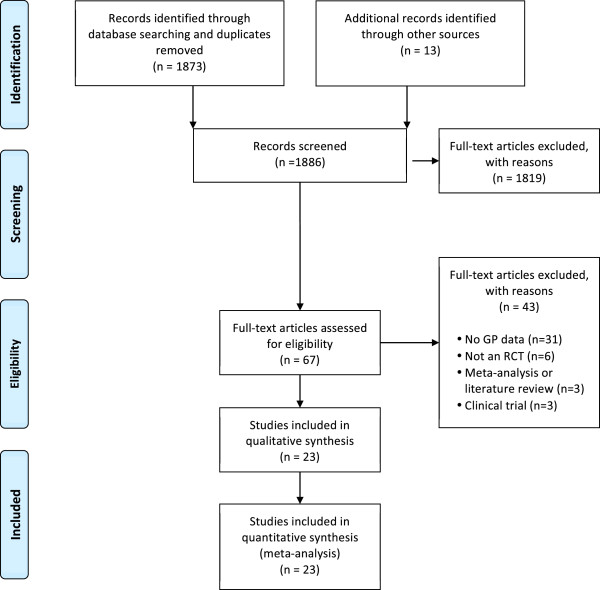
Flowchart.

Figure 1 displays the flowchart of the study selection process [20]. A total of 1873 records were identified through database searches. Reference lists of related literature and systematic reviews were also searched for additional papers [2,7,9,16,21-23]; Fourteen studies were identified, of which, one was a duplicate. Sixty-seven full-text articles were retrieved to determine eligibility. Forty-three studies were excluded with the following reasons: no GP data (n = 31), not an RCT (n = 6), meta-analyses or literature review (n = 3), clinical trial (n = 3) and two articles could not be retrieved. A total of 23 studies were included in this review.

We divided the strategies in two broad categories based on previous work conducted in this area: incentives and design based strategies [2]. There were 15 RCTs, four two-by-two factorial randomized design trials, one three-arm, two four-arm and one five-arm parallel randomized design trials (Table 1). There were three Australian, three American, one British and one Canadian study that measured the effectiveness of incentives. Nine Australian, three American, two Canadian, one Danish, and one Irish study included design based strategies. The majority of survey topics focussed on patient care, clinical guidelines and two studies [24,25] focussed on GPs personal work situation (eg job satisfaction). A summary of the risk of bias in included studies is presented in Table 2.

**Table 1:** Details of included studies

**Study ID**	**Aims of study**	**Study sample**	**Study topic**	**Study design**	**Total number of subjects in study (N)**	**Control (n)**	**Intervention (n)**	**Country**
Akl et al., 2011 [41]	Assess the effect of 1) tracking responses and 2) day of mailing (Monday vs Friday) on physician survey response rate	Directors of Family Medicine residency programs (ie GPs)	Training of residents in implementation of clinical guidelines	Two by two factorial randomized design	456	No tracking n = 228, Monday mailing n = 228	Tracking n = 228, Friday mailing n = 228	USA
Asch et al., 1998 [26]	To evaluate the effect of incentive size by providing physicians either a $5 bill or a $2 bill.	Primary care physicians identified from the American Medical Association Physician Master File	Attitudes about cost containment in cancer screening.	RCT	1000	$2 cash incentive received in initial mailing n = 500	$5 cash incentive received in initial mailing n = 500	USA
Baron et al., 2001 [29]	To evaluate the cost effectiveness of a lottery on physicians response rates to a mail survey.	Family Doctors identified from membership list of the Quebec Federation of GPs	The determinants of influenza immunization among family physicians in Quebec.	RCT	1000	No lottery n = 500	Lottery n = 500	Canada
Bonevskiet al., 2011 [40]	To examine the efficacy of two strategies for improving general practitioner response to a survey and a secondary aim was to assess GPs self reported preferred mode of survey administration.	GPs practising in NSW selected from the Australasian Medical Publishing Company database.	The knowledge, attitudes and practices of GPs around vitamin D.	RCT	334 (Cover letter trial), 1166 (Telephone reminder trial)	Standard cover letter N = 167, No telephone reminder n = 576	Standard letter plus local division of general practice cover letter n = 167, Telephone reminder n = 590	Australia
Deehan et al., 1997 [11]	To explore the effects of financial and non-financial inducements on response rates to increase the overall response rate.	A random sample of all GP’s in England and Wales.	Survey on GP’s work and attitudes with alcohol misusing patients.	Five arm parallel trial design with randomization	3584	No inducement n = 1188	£5 charity donation n = 607, £10 charity donation n = 578, £5cash payment n = 613, £10 cash payment n = 598, (Total for all interventions n = 2396)	UK
Drummond et al., 2008 [34]	To investigate the effect of two low cost interventions (pre-contact and questionnaire order) on response to a primary care physician postal survey	Primary care physicians working in Ireland identified from various sources.	A national survey assessing views and practices of physicians regarding prostate specific antigen (PSA) testing.	Two by two factorial randomized design	1599	No pre-contact n = 743, Version 2 survey (Topic specific questions first) n = 744	Pre-contact n = 715, Version 1 survey (general questions first) n = 714	Ireland
Everett et al., 1997 [28]	To examine the effectiveness of $1 incentive on response rates	Family Physicians	Firearm-safety counseling beliefs	RCT	600	No incentive n=300	$1 bill n=300	USA
Gattellari et al., 2012 [13]	Assess effectiveness of two response-aiding strategies in a postal survey	GPs known to be in current practice	Management of nonvalvular atrial fibrillation	Two by two factorial randomized control trial	1000	Mail prompt n = 500, Coloured seal without text n = 500	Fax prompt n = 500, Coloured seal + text n = 500	Australia
Gupta et al., 1997 [39]	To determine the effectiveness of a telephone prompt by a medical researcher compared to a nonmedical research assistant in improving survey response rate of GP’s, and to compare personnel costs.	A national random sample of Australian GPs	A national survey assessing GPs views of clinical practice guidelines	RCT	404	Telephone prompt by a medical researcher n = 202	Telephone prompt by experienced nonmedical research assistant n = 202	Australia
Hocking et al., 2006 [35]	To compare GP response to a telephone interview with response to a postal survey with three reminders.	A random sample of Victorian GPs	Survey to assess knowledge and diagnostic and management practices of genital chlamydia infection.	RCT	867	Postal survey n = 451	Telephone interview n = 416	Australia
James et al., 2011 [27]	To study the effects of payment timing, form of payment and requiring a social security number (SSN) on survey response rates.	Practising US physicians ages 65 and under representing all specialties (including primary care), selected from the AMA Physician Masterfile	Survey on ethical and moral beliefs.	Four arm parallel design with randomization	443	Promised $US25 check requiring SSN n = 102	Immediate $US25 cash n = 129, Immediate $US25 check n = 97, Promised $US25 check not requiring SSN n = 115	USA
McLaren and Shelley 2000 [30]	To compare effect of a primer telephone call and postcard on GP response rate; and to compare the effect of informing GPs about a prize for participating in survey compared to not informing GPs of the prize.	Victorian GPs	Management issues surrounding early pregnancy bleeding and miscarriage	Two by two factorial design with randomization	621	Telephone n=305 No prize notification N=310	Postcard n=316Prize notification n=311	Australia
Maheux et al., 1989 [42]	To determine the response rates of Quebec physicians by sending a handwritten thank you letter and by sending a more personalized mailout package.	Quebec Physicians	To determine the level of physicians support for a number of patient care issues.	RCT	Second mailing N = 356, Third mailing N = 253	No handwritten postscript n = 186, Non-personalised mailout n = 127	Handwritten postscript n = 170, Personalised mailout n = 126	Canada
Olivarius and Andreasen, 1995 [43]	To test a possible day the week effect on doctors response rate to a postal questionnaire.	GPs	A nationwide survey on doctors’ attitude on the relative importance of general practice and the specialties in the treatment of general diseases and diseases commonly regarded as belonging to the specialties.	RCT	200	Dispatch of survey on Thursday (for receipt before weekend) n = 98	Dispatch of survey on Saturday (for receipt after weekend) n = 102	Denmark
Pedrana et al., 2008 [44]	To compare response rates to postal surveys sent by normal post and registered mail and to assess the cost implications of the two mailing methods.	General practitioners in Victoria.	To assess antenatal screening practices in Australia	RCT	1550	Normal mail n = 775	Registered mail n = 775	Australia
Pirotta et al., 1999 [38]	To measure the effect of a primer postcard to improve mailed survey response rates.	Victorian GPs who had at least 1500 consultations in 1995-96	General Practitioners attitudes to complementary therapies.	RCT	800	No primer postcard n = 400	Primer postcard n = 400	Australia
Pit et al., 2013 [25]	To assess the effectiveness of small non-conditional non-financial incentive (ie attractive pen) on survey response rates	Practicing GPs in Northern Rivers region of NSW	How to improve sustainable employment of ageing GPs in rural Australia	RCT	125	No pen incentive n = 62	Pen incentive n = 63	Australia
Robertson et al., 2005 [31]	To test the effect of a $AUD2 scratch lottery ticket on response rates	A random sample GP’s and specialists from the medical directory of Australia.	To explore the uptake of 19 new drugs into the clinical practices of Australian GPs and specialists.	RCT	464	No lottery n = 232	AUD$2 scratch lottery n = 232	Australia
Scott et al., 2011[24]	To compare effects and costs of three different modes of survey administration in a national survey of Doctors	A stratified random sample of doctors undertaking clinical practice from a national directory of all doctors in Australia. Stratification was by doctor type and rural/remote category.	Study of the dynamics of the medical labour market in Australia, focusing on workforce participation and its determinants among Australian doctors.	3-arm parallel trial design with randomisation	1091	Online survey, followed by reminder letter with login details n = 369	Sequential mixed mode -online survey, followed by reminder letter with paper survey n = 388, Simultaneous mixed mode -paper survey and login details sent together, with reminder letter with login details n = 334	Australia
Seguin et al., 2004 [37]	To compare email with regular mail for conducting surveys of family physicians.	A random sample of physicians listed in the college of family physicians of Canada’s database who had an email address	Survey on use of sildenafil citrate (Viagra)	RCT	2397	Survey delivered by post to the physicians without email n = 800	Survey delivered by email n = 798, Survey delivered by post to the physicians with e mail n = 799	Canada
Shosteck and Fairweather, 1979 [36]	Comparison of mail and personal administration of questionnaire among physicians.	Office-based primary care physicians who were in active practice within the States of Washington DC and Maryland, and treating upper respiratory or lower urinary tract infections selected from the AMA database.	Physician antibiotic prescription practices.	RCT	543	Mail technique n = 296	Personal interview technique n = 247	USA
Thomson et al., 2004 [32]	To maximise the response to a postal questionnaire and to test the most effective form of incentive.	Practicing GP’s selected from the Lothian Primary Care NHS Trust database.	GP’s attitudes to the management of ingrowing toenails.	RCT	568	Lottery of one chance to win six bottles of champagne n = 286	Lottery of six chances to win one bottle of champagne n = 282	UK
Ward et al., 1998 [33]	To evaluate response aiding strategies feasible in large surveys	Randomly selected sample of general practitioners	Cancer screening	4-arm parallel trial design with randomisation	1550	-	Doctor advance phone prompt: n = 249, Pen prompt: n = 261, Letter prompt: n = 260, Non-doctor advance phone prompt: n = 501	Australia

**Table 2:** Quality assessment of included studies

**Study ID**	**Allocation concealed?**	**Randomisation performed?**	**Blinding of participants & personnel?**	**Complete outcome data?**	**Absence of reporting bias?**	**Absence of other bias?**	**ITT analysis?**
*Monetary incentives*							
1. Asch et al., 1998 [26]	?	√	?	√	√	√	√
2. Deehan et al.,1997 [11]	?	√	?	?	√	?	√
3. Everett et al.,1997 [28]	?	√	?	√	√	?	√
4. James et al., 2011 [27]	?	√	?	?	√	√	X
*Non-monetary incentives*							
5. Baron et al., 2001 [29]	?	√	?	?	√	√	√
6. McLaren and Shelley, 2000 [30]	?	√	?	?	√	?	X
7. Pit et al., 2013 [25]	X	√	X	?	√	√	√
8. Robertson et al.,2005 [31]	?	√	?	√	√	?	√
9. Thomson et al.,2004 [32]	?	√	?	?	√	?	√
*Questionnaire design/ mode of delivery*							
10. Drummond et al., 2008 [34]	√	√	?	√	√	√	√
11. Hocking et al., 2006 [35]	X	√	?	√	√	?	√
12. Shosteck and Fairweather,1979 [36]	?	√	?	√	√	√	√
13.Seguin et al.,2004 [37]	?	√	?	√	√	?	√
14. Scott et al., 2011 [24]	X	√	X	?	√	√	√
*Other design based strategies*							
15. Akl et al., 2011 [41]	X	√	?	?	√	?	√
16. Bonevski et al., 2011[40]	?	√	?	?	√	?	√
17. Gattellari et al., 2012[13]	X	√	X	?	√	√	√
18. Gupta et al.,1997 [39]	?	√	?	?	√	?	√
19. Maheux et al., 1989[42]	?	√	?	?	√	?	√
20. Olivarius and Andreasen, 1995 [43]	X	√	?	√	√	?	√
21. Pedrana et al.,2008 [44]	X	√	?	?	√	?	√
22. Pirotta et al.,1999 [38]	√	√	√	√	√	?	√
23. Ward et al., 1998 [33]	X	√	?	√	√	√	√

Legend:

?→Unclear.

X→No.

√→Yes.

### Incentives

#### Monetary incentives

Four studies examined the effects of monetary incentives on GPs’ response to surveys. Deehan and colleagues [11] found that cash payment and charity donation (£5 and £10) were more effective in increasing GP response rate compared to no incentive (pooled OR 1.87, 95% CI 1.48 to 2.36) (Table 3). The study also demonstrated that cash incentives were more effective than charity donations compared to no incentive, and larger cash incentives were more effective than smaller cash incentives.

**Table 3:** Monetary and non-monetary incentives

**Monetary incentives**	**Intervention**	**OR**	**95%CI**
Deehan et al., 1997 [11]	£10 vs no incentive	3.14	2.37, 4.15
	£5 vs no incentive	*2.22*	1.66, 2.98
	£10 charity donation vs no incentive	*1.20*	0.85, 1.69
	£5 charity donation vs no incentive	1.12	0.79, 1.57
	*Pooled (Deehan, 1997)*	*1.87*	*1.48, 2.36*
Everett et al., 1997 [28]	$1bill vs no incentive	1.86	1.34, 2.57
	*Pooled*	*1.87*	*1.55, 2.26*
**Nonmonetary incentives**			
Baron et al., 2001 [29]	Lottery to win weekend trip vs No incentive	1.31	1.02, 1.70
McLaren and Shelley, 2000 [30]	Awareness of prize vs no awareness of prize	1.17	0.84, 1.61
Pit et al., 2013 [25]	Pen vs no incentive	1.85	0.91, 3.77
Robertson et al., 2005 [31]	AU$2 Lottery vs no incentive	1.78	1.23, 2.60
	*Pooled*	*1.39*	*1.15, 1.69*
**Large vs small incentives**			
Asch et al., 1998 [26]	US$5 vs US$2	1.83	1.43, 2.35
Deehan et al., 1997 [11]	£10 cash vs £5 cash	1.41	1.06,1.87
	£10 charity donation vs $5	1.08	0.73,1.58
Thomson et al., 2004 [32]	Lottery to win a large prize (6 bottles of champagne) vs six chances to win one bottle	1.47	1.05, 2.08
	*Pooled*	*1.47*	*1.19,1.81*
**Prepaid vs promised incentives**			
James et al., 2011 [27]	US$25 prepaid vs US$25 promised	2.88	1.70, 4.89

Asch and colleagues [26] also found that a larger cash incentive (US$5) was more effective than a smaller cash incentive (US$2) (OR 1.83, 95% CI 1.4 to 2.35). Everett and co-workers found that a $1 bill was more effective than no incentive. James and colleagues [27] found that upfront payment of a cash incentive (US$25) was significantly more effective than a promised cash incentive (US $25) (OR 2.88, 95% CI 1.70 to 4.89).

The extent of the risk of bias in all four studies examining monetary incentives is unclear as there was insufficient detail to determine if allocation of subjects were concealed, if participants and personnel were blinded to the study. In two studies it was not clear if there was absence of other bias. Missing data due to loss of follow up (eg undelivered surveys) was equally distributed between study groups in two studies [26,28], but this information was not reported in the other two studies [11,27]. Only James and co-workers [27] did not undertake an intention to treat analysis, which may have over estimated the effect of the intervention.

#### Nonmonetary incentives

Four studies [25,29-31] examined the effects of nonmonetary incentives compared to no incentives on GP response rate to surveys. The weighted overall effect size showed a small but significant association between nonmonetary incentives and GP response (OR 1.39, 95% CI 1.15 to 1.69). Thomson et al. [32] compared two types of lottery incentives, and found that a lottery for one chance to win a large prize (6 bottles of champagne) was more effective than six chances to win one bottle (OR 1.47, 95% CI 1.05 to 2.08). All five studies that assessed nonmonetary incentives were randomised control trials, four undertook intention to treat analysis and all reported on pre-determined primary outcomes. The adequacy of allocation concealment and blinding of participants and personnel are unclear in four studies. Four studies did not report missing data due to loss to follow up, whilst only Robertson et al. [31] reported that missing data were equally distributed between study groups. No significant differences in demographic characteristics between study groups were found in Baron et al. [29] and Pit et al. [25], whilst this risk of bias was not clear in Robertson et al. [31], Thomson et al. [32] and Mclaren and Shelley [30].

#### Size of incentives

Three studies[11,26,32] found that a larger incentive had a small but significant effect on response rates (OR 1.47, 95% CI 1.19 to 1.81).

Finally, Ward and co-workers [33] used a pen as an incentive and compared it with other recruitment methods but did not find that a pen increased the response rates. They did find that women were more likely to respond to a pen.

### Design based strategies

One study [34] found that asking general questions first do not significantly increase GP survey response rates.

Three studies [35-37] demonstrated that postal surveys are significantly more effective than telephone or email surveys (OR 1.82, 95% CI 1.19 to 2.78). Scott and colleagues [24] found that an online survey did not lead to increased response rates compared to using mixed methods. However, they found that sending a letter containing login details and an option to request a paper copy followed by a reminder that included login details and a paper copy led to increased response rates when compared to online surveys.

Two studies [34,38] found that pre-contact with GPs via a postcard or letter compared to no intervention increased the response rate but this was just not statistically significant (OR 1.16, 95% CI 0.99 to 1.37).

In terms of mode of the pre-contact, Gattellari et al. [13] found that pre-contact by fax was not statistically different to pre-contact by mail in increasing GP response rates. Gupta et al. [39] found that there was no statistical difference between pre-contact by a medical researcher compared to a non-medical researcher. But a study conducted by Ward and colleagues [33] demonstrated that an upfront phonecall from a peer led to increased response rates when compared to 3 other methods (pen, letter and research assistant prompt).

Bonevski and co-workers [40] found that reminder telephone calls to non-responders prior to the 3^rd^ mailout did not significantly increase response rates when compared to not conducting reminder telephone calls. Akl and co-workers [41] found that tracking of responses did not help to improve response rates over not tracking responses. (Table 4)

**Table 4:** Design based strategies

**Questionnaire design**	**Intervention**	**OR**	**95% CI**
Drummond et al., 2008 [34]	General questions vs topic related questions first	1.17	0.96, 1.43
**Survey delivery mode**	**Intervention**		
Hocking et al., 2006 [35]	Postal survey vs telephone interview	2.66	2.02, 3.52
Shosteck and Fairweather, 1979 [36]	Postal survey vs telephone interview	1.03	0.68, 1.55
Seguin et al., 2004 [37]	Postal survey vs email survey	2.00	1.67, 2.38
	*Pooled*	*1.82*	*1.19, 2.78*
Scott et al. 2011 [24]	Online survey vs mixed mode	0.73	0.51, 1.04
	Online survey vs sequential mixed mode	0.65	0.44, 0.97
	Online survey vs Simultenous mixed mode	0.83	0.55, 1.26
**Advance contact**			
Drummond et al., 2008 [34]	Letter pre-contact letter vs no pre-contact	1.10	0.91, 1.35
Pirotta et al., 1999 [38]	Postcard pre-contact vs no pre-contact	1.30	0.98, 1.72
	*Pooled*	*1.16*	*0.99, 1.37*
Gattellari et al., 2012 [13]	Mail pre-contact vs fax pre-contact	1.11	0.87, 1.43
McLaren and Shelley, 2000 [30]	Telephone pre-contact vs postcard pre-contact	1.01	0.73, 1.40
Gupta et al., 1997 [39]	Medical researcher pre-contact vs non-medical researcher	1.33	0.87, 2.05
Ward et al., 1998 [33]	GP phonecall pre-contact vs other interventions	1.39	1.02, 1.88
	Pen pre-contact vs other interventions	0.97	0.73, 1.30
	Letter pre-contact vs other interventions	0.95	0.71, 1.26
	Research assistant phonecall pre-contact vs other interventions	0.86	0.68, 1.09
**Reminders**			
Bonevski et al., 2011 [40]	Telephone reminder vs none	1.21	0.68, 2.15
**Tracking**			
Akl et al., 2011[41]	Tracking of reponses for follow up vs No tracking	0.85	0.59, 1.23
**Personalisation and GP group endorsed letters**			
Bonevski et al., 2011[40]	Sponsorship letter vs standard letter 1^st^ mailout	1.38	0.86, 2.22
Gattellari et al., 2012 [13]	Coloured seal with text vs coloured seal without text	1.08	0.84, 1.38
Maheux et al., 1989[42]	Handwritten ‘thank you’ postscript with 1st reminder vs no postscript	1.45	0.92, 2.30
Maheux et al., 1989[42]	Personalised mail out package vs Non-personalised mail out package with 2^nd^ reminder	1.73	1.04, 2.87
**Timing of mailing**			
Akl et al., 2011[41]	Friday vs onday mailing	1.86	1.28, 2.69
Olivarius and Andreasen, 1995 [43]	Saturday vs Thursday mailing	0.55	0.25, 1.23
	*Pooled*	*1.07*	*0.33, 3.50*
**Type of mailing**			
Pedrana et al., 2008 [44]	Registered mail vs standard mail	2.97	2.31, 3.81

Personalisation of mail-out packages to GPs generally does not appear to increase response rates. Inclusion of a professional sponsorship letter, coloured seal with text and hand written thank you postscripts did not increase survey response rates [13,40,42]. However, Maheux et al. [42] found that a personalised mail-out package that included the physician’s title, name and address individually typed onto the envelope, hand stamped outgoing envelopes identified to the university and hand stamped return envelope increased response rates with modest statistical significance (OR 1.73, 95% CI 1.04 to 2.87).

Akl and colleagues [41] found that Friday morning was more effective than a Monday mailing. However, the pooled results of Akl et al. [41] and Olivarius and Andreasen [43] showed that timing of mailing at the end of the week compared to during the week does not effect survey response rates amongst GPs. Pedrana and co-workers [44] found that surveys sent by registered post significantly increased the response rates compared to surveys sent by standard post (Table 4).

The majority of included studies that assessed the impact of design based strategies reduced the risk of bias by randomisation, reporting of predetermined primary outcomes and intention to treat analysis. A high risk of selection bias due to inadequate allocation concealment was evident in half (7 of 14) of these studies. Blinding of participants and personnel was undertaken in only one study, not undertaken in two of the studies and unclear in 11 studies.

Missing data were equally distributed between study groups in seven studies and unclear in the remaining seven studies. The balance of demographic characteristics between study subjects was demonstrated in five studies and unclear in the other eight studies (Table 2).

## Discussion

This review identified several strategies that can easily be implemented by researchers and policy makers to increase response rates for GP surveys. Monetary and nonmonetary incentives were more effective than no incentive. Large incentives were more effective than small incentives as were upfront monetary incentives compared to promised monetary incentives. Postal surveys were more effective than telephone or email surveys. One study demonstrated that sequentially mixed mode (online survey followed by a paper survey with the reminder) was more effective than an online survey or the combination of an online and paper survey sent similtaneously in the first mail-out. Pre-contact with a phonecall from a peer colleague, personalised packages, sending mail on Friday, and using registered mail also increased response rates in single studies. Pre-contact with a letter or postcard increased reponse rates slightly but just did not reach statistical significance.

This review focussed specifically on GPs to assist in increasing GP survey response which limits its generalisability to other doctor groups. There is evidence that GPs and specialists respond differently to recruitment strategies. For example, Robertson and colleagues found that incentives had a quantitatively larger effect amongst GPs compared to specialists response rates but this was not statistically significant [31]. Scott and co-workers [24] found that GPs had the lowest response rates and specialists the highest response rates. Specialists had statistically significant higher response rates compared to other doctors when offered an online and paper survey at the same time. On the contrary, Olivarius and Andreasen [43] concluded that GPs had higher response rates than specialists. Furthermore, Maheux and colleagues [42] reported that specialists were a little more sensitive to the interventions than the GPs. Lastly, James and colleagues found that there was no difference between doctor types and the impact of incentive type and timing on survey response rates [27]. The difference in reponse rates per type of doctor may well depend on a variety of factors including socio-demographic factors, the topic of interest and the level of involvement required to participate in the survey. This should be taken into account when designing recruitment strategies for surveys.

We found substantial heterogeneity among the included studies which makes it difficult to generalise the study findings and determine the impact of other factors on the study results [3]. For example, in the general population more sensitive topics lead to lower survey response rates [8]. This was difficult to assess in this systematic review because there were not any study topics that were considered to be sensitive for GPs. In the study published by Seguin and colleagues in 2004 [37], the difference in response rate between postal and email surveys could be attributed to the year that the study was conducted because GPs may now be more used to email. A more recent study [24] found that online surveys led to lower response rates than mixed mode approaches (both online and paper based surveys). The reluctance of GPs to complete surveys online is confirmed in other studies [40]. Potential explanations are concerns about confidentiality, familiarity and access to the internet, and the ease of completing the survey online ( eg the need to recall or enter a password). It is possible that online response rates may improve in the future because of the growing use of smartphones and portable devices that make it easier and quicker to access online surveys. The number of reminders also may have had an impact on study results. The largest impact of incentives and other recruitment strategies is often seen in the first wave of recruitment and this steadily decreases with followup reminders [25,26].

Researchers must take into account the available resources when designing survey recruitment strategies. Shorter surveys, online surveys, the number of reminders and smaller incentives can all lead to cost-savings but this needs to be carefully balanced with maximising high quality, valid and reliable data. Eleven studies in this systematic review included information on intervention costs [24,26,29-31,34,36,38,39,41,44]. Three out of four studies which used nonmonetary incentives provided relevant cost information. Baron et al. [29], Mclaren and Shelley [30] and Robertson et al. [31] found that by offering a nonmonetary incentive at an additional cost of CAN$16, AU$3.46 and AU$23.42 per survey returned led to an increased response rate of respectively 6%, 4% and 15%. Asch and co-workers [26] found that a $5 incentive versus a $2 incentive cost only an additional $0.53 cents per survey returned with an increase in response rates of 15%.

Amongst design based studies, Scott and colleagues [24] found that the additional cost per 1% increase in response rate was AU$3290 for sequential mixed mode strategies and AU$10,156 for simultaneous mixed mode strategies when compared to the online mode. The additional cost per additional response was AU$6.07 for sequential mixed mode strategies and AU$18.75 for simultaneous mixed mode strategies with an increase in GP response rates of 6% and 2% respectively when compared to the online survey (response rate 14%). The sequential mixed mode strategy therefore appears to be the most cost-effective method. However, these cost figures need to be weighed up against the fact that there was some response bias in the sequential and simultenous mixed mode strategies and a higher rate of item non-response in the online survey. Researchers should be aware that there is some evidence that there are new differences between physician responders and non-responders and early and late responders, suggesting that there is a low level of nonresponse bias [2,14]. It is argued that the reason for this is that the population is rather homogeneous.

Little differences in cost per respondent were found in studies that used a letter or postcard as a pre-contact strategy. Drummond and co-workers [34] reported that sending a postcard led to an additional cost of â‚¬0.87 per respondent and an increase in the reponse rate of 4%. The non-postcard group had a response rate of 46% compared to 50% in the postcard group. Similarly, Pirotta et al. [38] found that sending a postcard added only AU$0.40 per respondent but increased the reponse by 6% from 60% to 66%. McLaren and co-workers [30] found no difference between telephone pre-contact and postcard pre-contact but that the costs involved in calling GPs was about 5 times as much as sending a postcard. Gupta and colleagues [39] found that pre-contact by a medical practitioner and a research assistant led to the similar costs because the research assistant had to make more calls and there was no statistical significant difference in response rates. It therefore appears that pre-contacting GPs by mail is a cheap method to improve response rates compared to doing nothing, and is cheaper than telephone pre-contact which may lead to similar response rates. Lastly, Pedrana and co-workers [44] found that sending surveys by registered mail cost an additional AU$1531.50 but led to a 19% increase in response rates (response rate: 86% registered mail versus 67% normal mail.). This seems to suggest that extra costs are justified in these cases.

In accordance with our study, systematic review studies amongst physicians have also found that monetary incentives increase response rates [2,14], as does pre-payment [2,14]. Contrary to our findings, Van Geest and Kellerman found that non-monetary incentives did not increase response rates amongst physicians [2,14]. This may also reflect differences between GPs and other physicians. VanGeest et al. [2] pointed out that non-monetary incentives are more likely to work if physicians value them. Large incentives were more effective than small incentives in our study and Kellerman and Herold's study [14]. However, Vangeest found that larger incentives led to mixed results for both monetary and non-monetary incentives [2]. Kellerman and Herold [14] found no difference between mail and telephone interviews, whereas our review did. This may be explained by the fact that our review included more recent studies. VanGeest also reported that family physicians prefer mail surveys [2]. The general consensus is that GPs still prefer mail surveys over online surveys. Pre-contact with a phonecall from a peer colleague, personalised packages, sending mail on Friday, and using registered mail also increased response rates in single studies. Kellerman and Herold [14] found that pre-notification was not effective but this conclusion was based on only one study. Kellerman and colleagues reported one study that found that personalised mailouts were effective during the 1^st^ mail out but no effect was found during 2^nd^ mailouts in two other studies. However, Maheux et al. [42] demonstrated an effect of personalised mailouts during 3^rd^ mailouts. This effect is likely to be explained by the fact that the researchers included multiple elements to personalise the package.

The quality of the included studies generally was not clear from current reporting. Allocation concealment and blinding of participants were clearly reported in only two studies. Complete outcome data were available for 10 studies. Absence of reporting bias was present in all studies, whereas absence of other bias was only applicable to 9 studies. The strength of this review is that , to our knowledge, this is the first systematic review that has examined GPs as a group and not a sample of multiple different medical practitioners. Another strength of this review is that the outcome measure is an objective outcome measure and is therefore less likely to be influenced by reporting bias within studies. A weakness of the study was that we did not approach authors for additional information due to the budget constraints. This has an impact on judging the quality assessment of the included studies as mentioned above, in particular in the area of blinding of participants and personel. Finally, a limitation of the study is that the majority of studies were conducted in English speaking countries and may therefore limit generalsibility to other countries.

Further research is required to advance this field of research. Areas that need further exploring include strategies and factors such as survey topic, confidentiality guarantees, level of incentives, use of social media, mobile phone applications, and sequential mixed method applications. Furthermore, better reporting is required to determine the quality of the included studies. We support Creavin and colleagues’ suggestion to develop a standard template for survey studies, similar to the consort statement [3] to improve reporting on survey methodology. Finally, we also recommend that future studies measure and report on the resources used when conducting the interventions to guide future researchers in strategy selection. We recommend that primary care researchers can build randomised controlled trials into survey research to further test which strategies are most effective.

## Conclusions

GPs response rates to surveys may improve by using the following strategies: monetary and nonmonetary incentives, larger incentives, upfront monetary incentives, postal surveys, pre-contact with a phonecall from a peer colleague, personalised packages, sending mail on Friday, and using registered mail. Mail pre-contact may also improve response rates and has low costs. Improved reporting and further trials, including mixed mode studies, are required to determine the effectiveness of recruitment strategies on general practitioners’ response rates to surveys.

## Competing interests

All authors have completed the Unified Competing Interest form at http://www.icmje.org/coi_disclosure.pdf (available on request from the corresponding author) and declare that none of the authors (SWP, TV or SP) have no non-financial interests that may be relevant to the submitted work. All authors declare they have no competing interests.

## Authors’ contributions

SWP designed the study, carried out the searches, screened and data-extracted studies, analysed the data and interpreted study results, and prepared the manuscript. She is the guarantor. TV data-extracted studies, analysed the data and helped prepare the manuscript. SP assisted in searches, screened studies and data-extracted studies. All authors read and approved the final manuscript.

## Pre-publication history

The pre-publication history for this paper can be accessed here:

http://www.biomedcentral.com/1471-2288/14/76/prepub

## Supplementary Material

Additional file 1 Search: Medline strategy. Click here for file
