# Supplementary material for: The effectiveness of recruitment strategies on general practitioner’s survey response rates – a systematic review
Source: BMC Med Res Methodol. 2014 Jun 6;14:76. doi: 10.1186/1471-2288-14-76 (PMC4059731; doi:10.1186/1471-2288-14-76)
Supplement: Additional file 1 — Search: Medline strategy. [file 1471-2288-14-76-S1.pdf]

Additional files

Additional file 1 – Search: Medline strategy

1. FAMILY PRACTIC/ or family practi$.tw.
2. general practice$.tw.

3. PRIMARY HEALTH CARE/ or primary health care.tw.

4. primary care$.tw.

5. PHYSICIANS, FAMILY/

6. (family physician$ or family doctor$).tw.

7. (GP$ or GP$).tw.

8. (Clinician$ or physician$ or practitioner$ or surgeon$).tw.

9. or/1-8

10. (remind* or letter* or postcard* or incentiv* or reward* or money* or monetary or payment* or lottery or raffle or prize or personalis* or sponsor* or anonym* or length or style* or format or appearance or color or colour or stationary or envelope or stamp* or postage or certified or registered or telephon* or telefon* or notice or dispatch* or deliver* or deadline or sensitive or disseminate).tw.

11. telephone/

12. cellular phone/

13. (telephon$ or telefon$).tw.

14. phone.tw.

15. ((mobile adj10 $phone) or (cell adj10 $phone)).tw.

16. or/11-15

17. Internet/

18. Online Systems/

19. Electronic Mail/

20. (email or e*mail).tw.

21. (internet or online or webcam or skype or messenger or chat).tw.

22. or/17-21

23. (Post* or mail*).tw.

24. Telefacsimile/

25. (Fax or facsimile).tw.

26. or/24-25

27. exp Interview/

28. Interview*.tw.

29. or/27-28

30. (questionnair* or survey* or data collection or instrument* or tool*).tw.

31. Questionnaires/

32. or/30-31

33. 10 or 16 or 22 or 23 or 26 or 29 or 32

34. ((Respons* or cooperation or consent or contact or refusal) adj1 $rate).tw.

35. (respon* or return*).tw.

36. (item or items or question or questions or answer or answers or response or responses).tw.

37. (nonrespond* or non-respond* or nonrespons* or non-respons* or missing or missed or omission or omitted or incomplete* or bias* or complete* or inconsist* or (data and quality) or incorrect*).tw.

38. (follow adj up).tw.

39. (Enhanc* or improv* or promot* or increas* or influenc* or maximi*).tw.

40. or/34-39

41. 9 and 33 and 40

42. Rural Health Services/

43. Medically Underserved Area/

44. Rural Areas/

45. Hospitals, Rural/

46. Rural Health Centers/

47. ((rural or remote or nonmetropolitan) adj (health service? or health care or healthcare or medical service? medical care)).tw.

48. ((rural or remote or nonmetropolitan or underserved or under served or deprived) adj (communit$ or area? or region? or province?)).tw.

49. (rural adj (setting? or clinic? or hospital?)).tw.

50. shortage area?.tw.

51. (inequitable distribute$ or maldistribut$).tw.

52. or/42-51

53. Rural Health Personnel/

54. Rural Health Nursing/

55. 52 or 53 or 54

56. randomized controlled trial.pt.

57. controlled clinical trial.pt.

58. randomized.ab.

59. placebo.ab.

60. clinical trials as topic.sh.

61. randomly.ab.

62. trial.ti.

63. or/56-62

64. exp animals/ not humans.sh.

65. 63 not 64

66. 9 and 33 and 40 and 6
